# Supplementary material for: Levels of Cell-Free DNA in Kidney Failure Patients before and after Renal Transplantation
Source: Cells. 2023 Dec 6;12(24):2774. doi: 10.3390/cells12242774 (PMC10741614; doi:10.3390/cells12242774)
Supplement: Supplementary file 1 [file cells-12-02774-s001.zip › cells-2718609-supplementary.pdf]

SUPPLEMENT FIGURES

Supplement Table S1 - Clinical profile of HD and non-HD patients before KT

	**HD (N=22)**	**Non-HD (N=22)**	***p*-value**
Age, years	47 (32 – 50)	44 (30-49)	0.3417
BMI, kg/m^2	23.4 (20.9 – 28.1)	22.8 (21 – 26.6)	0.6886
Males	17 (77)	16 (73)	0.7277
SBP, mmHg	141 (124 – 161)	137 (128 – 162)	0.8027
DBP, mmHg	86 (75 – 95)	90 (79 – 96)	0.3723
CVD	17 (77)	22 (100)	**0.017***
DM	2 (9)	1(5)	0.5498
Medications at cohort entry			
ACE-inhibitors/ARBs	13 (59)	12 (55)	0.7609
Beta-blockers	16 (73)	14 (67)	0.6653
Ca^{2+} channel blockers	10 (45)	16 (73)	0.0658
Statins	4 (18)	5 (23)	0.7086
Biochemicals			
Cholesterol, mmol/L	4.5 (3.6 – 5.2)	4.5 (4.0 – 4.9)	0.9305
HDL, mmol/L	1.4 (1.1 – 1.5)	1.4 (1.0 – 1.7)	0.7657
Triglycerides, mmol/L	1.3 (1.0 – 1.6)	1.4 (1.1 – 1.8)	0.3529
Lp (a), mg/L	83 (42 – 154), n = 10	43 (11 - 93)	0.1015
Apo-A1, g/L	1.4 (1.2 – 1.6)	1.4 (1.3 – 1.6)	0.6377
Apo-B, g/L	0.8 (0.7 – 1.0)	0.9 (0.8 – 1.0)	0.8207
Creatinine, mg/dL	7.7 (6.3 – 9.9)	7.0 (6.4 - 9.7)	0.6672
Albumin, g/L	36 (33 - 40), n = 21	36 (32-38)	0.5662
HbA1c, %	5.2 (4.9 – 5.5)	5.3 (5.1 – 5.6), n = 21	0.4006
hs-CRP, mg/L	1.6 (0.4 – 2.9)	0.8 (0.3 – 1.6)	0.2132
25 (OH) vitD, nmol/L	45 (37.3 – 70.8)	53 (30.5 – 65.5)	0.8388
Homocysteine, µmol/L	34 (23.8 – 47.5)	38 (30.8- 60.5)	0.1069
Calcium, mmol/L	2.3 (2.1 – 2.4), n = 21	2.4 (2.1 – 2.4)	0.7959
P-Phosphate, mmol/L	1.4 (1.2 – 1.8), n =21	1.7 (1.5 – 1.9)	0.3290
Folate, nmol/L	13 (9 - 45), n = 21	10 (6 – 15)	0.6153
P-Troponin T, µg/L	24 (17.5 – 48.5), n = 21	17.5 (12.8 – 23.8)	**0.0371***
Calprotectin, µg/ml	2.4 (1.7 – 3.3), n = 19	2 (1.4 – 3.1), n = 20	0.3259
MMP-9, ng/mL	362 (286 - 451), n = 17	439 (312-696), n=17	0.3571

Data are presented as median and interquartile range (Q1-Q3). Categorical data are presented as frequency (%). Continuous data were analysed by non-parametric Mann-Whitney test. Categorical data were analysed by χ^2. Significance was established at *p<0.05.

Abbreviations: BMI – body mass index; SBP - systolic blood pressure; DBP – diastolic blood pressure; CVD – cardiovascular disease; DM – diabetes mellitus; HD – haemodialysis; eGFR – estimated glomerular filtration rate; ACE – angiotensin converting enzyme; ARB – angiotensin receptor blocker: Ca – calcium; HDL – high density lipoprotein; Lp(a) – lipoprotein(a); Apo-A1 – apolipoprotein -A1; Apo-B – apolipoprotein – B; HBA1c – haemoglobin A1c; hsCRP – high sensitivity C-reactive protein; MMP – 9; matrix metalloprotein 9.

Supplement Table S2 - Correlation of cfDNA fractions in HD and non-HD patients at basal and 2 years post-KT

	HD			
	**Basal (n=22)**		**2 years post KT (n=22)**	
	r	*p* value	r	*p* value
**total cfDNA**				
hs-CR P,mg/L	0.084	0.711	0.477	**0.025***
Apo-B, g/L	-0.579	**0.005***	-0.002, n=20	0.995
Vintage years	0.470, n=18	**0.049***	--	--
**mt-cfDNA**				
Triglycerides, mmol/L	0.530	**0.011***	0.130, n=20	0.584
Albumin, g/L	0.486, n=21	**0.025***	0.105, n=21	0.652
**nc-cfDNA**				
Triglycerides, mmol/L	0.432	**0.044***	0.196, n=20	0.408
Apo-B, g/L	-0.437	**0.042***	0.298, n=20	0.202

	Non-HD			
	**Basal (n=22)**		**2 years post KT (n=22)**	
	r	*p* value	r	*p* value
**total cfDNA**				
MMP-9, ng/mL	-0.608, n=17	**0.010***	--	--
**mt-cfDNA**				
Lp (a), mg/L	0.516	**0.014***	0.330	0.167
Homocysteine, μmol/L	0.455	**0.033***	-0.095	0.690
BMI, kg/m^2	-0.482	**0.023***	--	--
**nc-cfDNA**				
Creatinine, mg/dL	0.503	**0.017**	-0.064	0.778
MMP-9, ng/mL	-0.495	**0.043**	--	--

Correlations were assessed using non-parametric Spearman's rank correlation for each laboratory measurement. Significant result was established at *p* value <0.05.

Abbreviations: HD – haemodialysis; KT – kidney transplantation; cfDNA – cell free DNA; hsCRP – high sensitivity C-reactive protein; apo-B – apolipoprotein-B; MMP9 – matrix metalloprotein 9; Lp(a) – lipoprotein A; BMI – body mass index

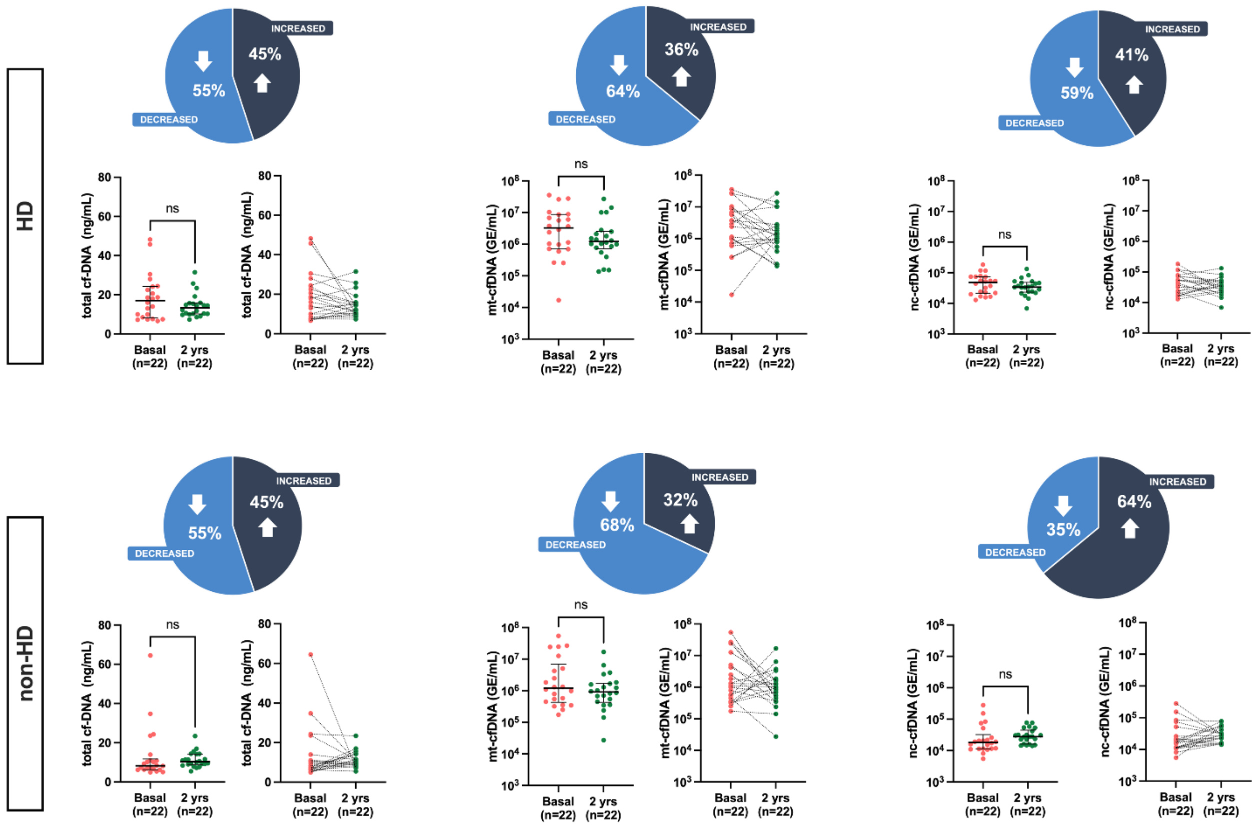

Supplement Figure S1. Measurement of cfDNA levels at baseline and two years post-KT among patients who received HD and conservative therapy (non-HD). Data is presented as median and interquartile range (Q1-Q3). Statistical significance, *p<0.05.

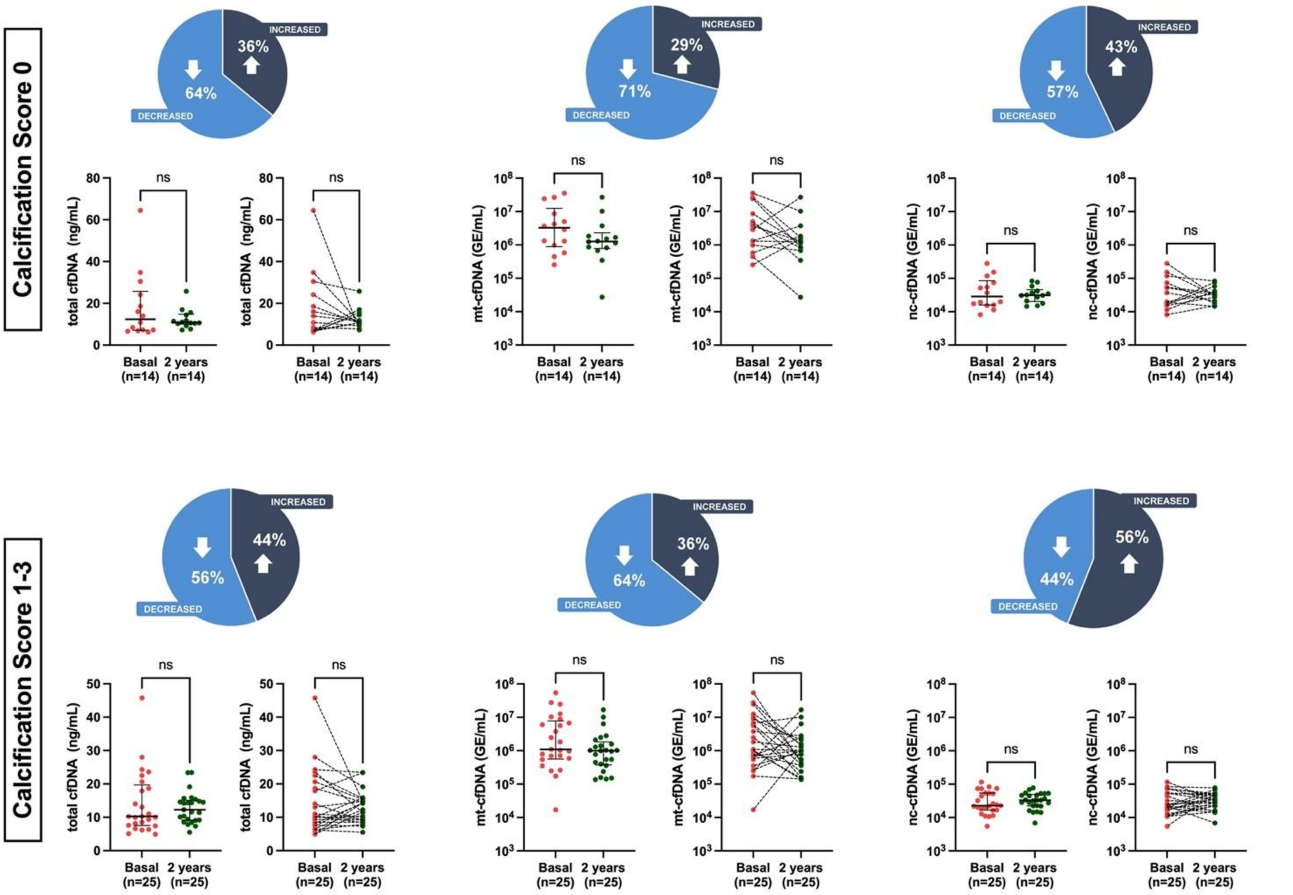

Supplement Figure S2. Measurement of cfDNA levels according to Calcification Scores. Data is presented as median and interquartile range (Q1-Q3). Statistical significance, *$p<0.05$

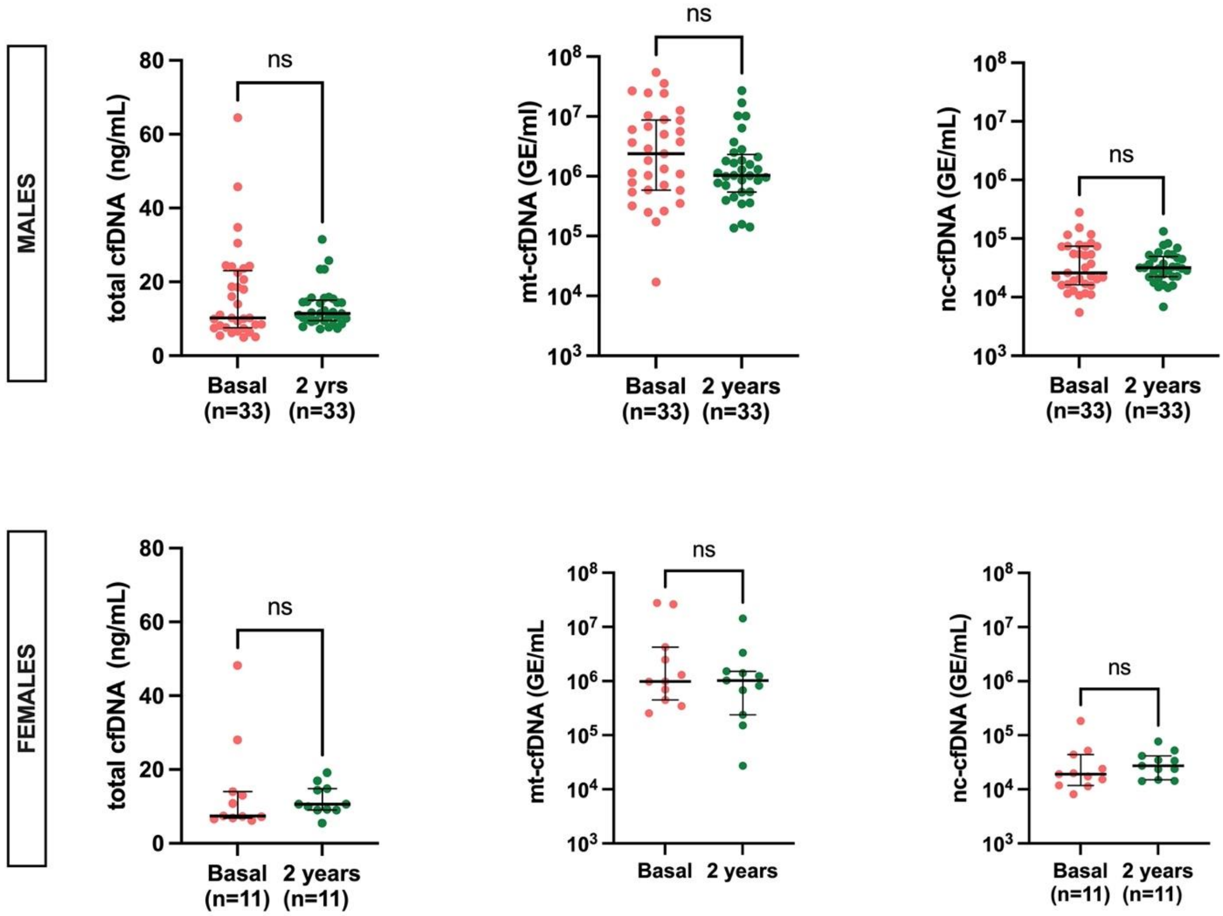

Supplement Figure S3. Measurement of total cfDNA, mt-cfDNA and nc-cfDNA levels among male and female patients at baseline vs 2 years after KT. Data is presented as median and interquartile range (Q1-Q3). Statistical significance, *p<0.05.

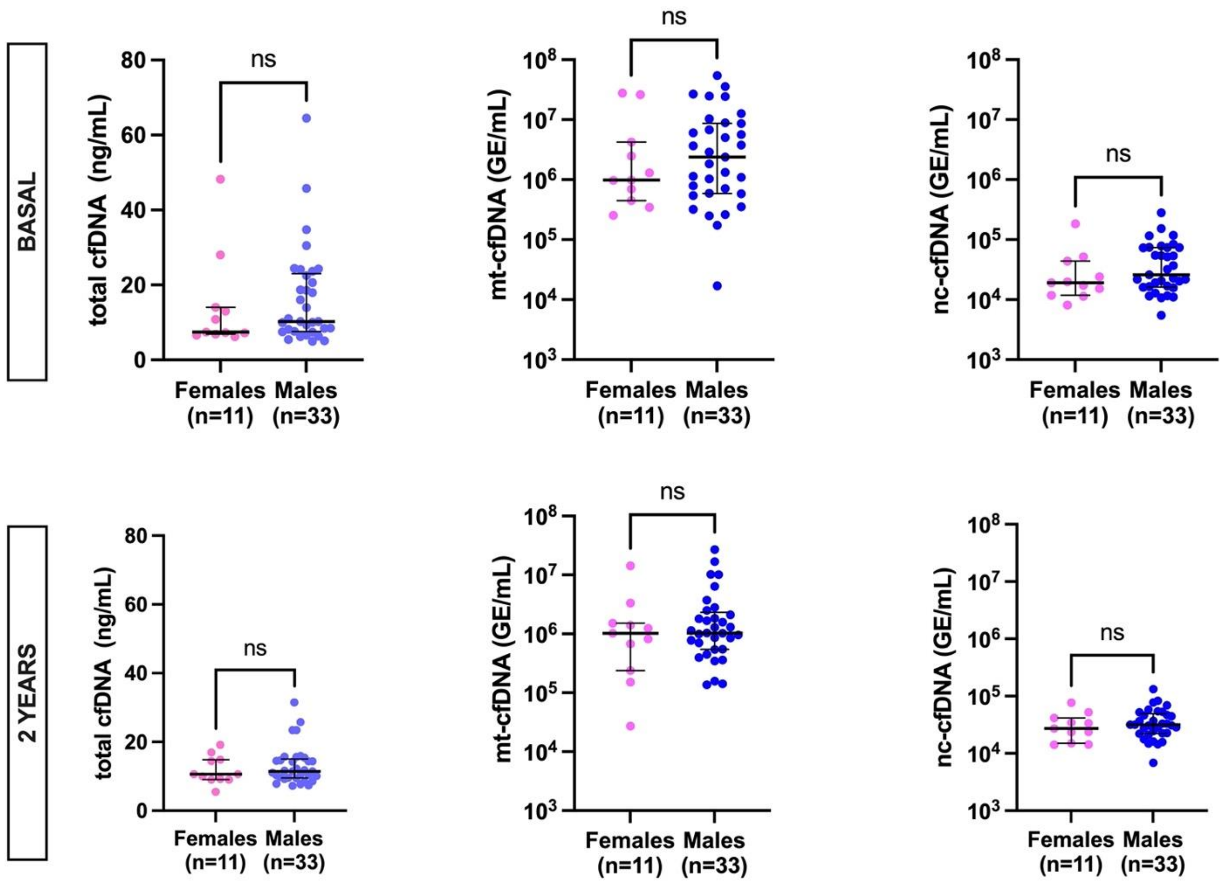

Supplement Figure S4. Measurement of total cfDNA, mt-cfDNA and nc-cfDNA levels at baseline and 2 years post KT among female vs male patients. Data is presented as median and interquartile range (Q1-Q3). Statistical significance, **p*<0.05.
